# Development of a Novel Immunoprecipitation Method for Extracting Monoclonal Antibodies From Brain Tissue and Its Application to Assessing In Vivo Brain Penetration in Mouse via Liquid Chromatography–Mass Spectrometry

**DOI:** 10.1002/bmc.70196

**Published:** 2025-08-14

**Authors:** Sangsoo Hwang, Seo‐jin Park, Jeong‐hyeon Lim, Young G. Shin

**Affiliations:** ^1^ Institute of Drug Research and Development, College of Pharmacy Chungnam National University Daejeon South Korea

## Abstract

A novel extraction method was developed to quantify monoclonal antibodies from brain tissue, crucial for pharmacokinetic assessments. We focused on optimizing detergent use for compatibility with liquid chromatography–mass spectrometry analysis. IGEPAL and sodium deoxycholate were selected for their ability to preserve antibody integrity during extraction, with conditions optimized to a 4% concentration. This was most effective for maintaining antibody structure and maximizing recovery. Pharmacokinetic analysis was conducted on the 8D3 monoclonal antibody, known for its brain penetration abilities. Administered at 20 mg/kg, its concentration in the brain was measured using the optimized extraction methods. The 4% SDC method showed superior extraction efficiency, significantly enhancing brain exposure levels with a higher maximum concentration and area under the curve compared to the 4% IGEPAL and detergent‐free methods. This highlights the importance of detergent selection in accurate pharmacokinetic measurements. We also addressed potential blood contamination. Specific perfusion conditions effectively removed 98% of blood contaminants, ensuring that pharmacokinetic measurements reflected true monoclonal antibody levels in brain tissue. Overall, this study establishes a robust method for monoclonal antibody extraction from brain tissue, enhancing the analysis of brain‐targeted therapies and providing a valuable framework for future studies aiming to measure drug concentrations in brain tissue.

## 1 Introduction

Recent advances in biological therapeutics, particularly monoclonal antibodies, have introduced novel platforms for treating central nervous system (CNS) disorders (Tambuyzer et al. 2020). Despite promising in vitro results, these agents often exhibit poor in vivo efficacy due to the restrictive nature of the blood–brain barrier (BBB) (Fricker and Mahringer 2010). To address this, receptor‐mediated transcytosis (RMT) strategies have been developed to utilize endogenous transport mechanisms at the BBB. Therapeutic molecules engineered to bind specific receptors on the luminal surface of brain endothelial cells can be actively transported across the barrier into brain tissue via vesicular trafficking (Haqqani et al. 2024). Among the RMT targets, the transferrin receptor (TfR) has been widely studied (Bien‐Ly et al. 2014; Pardridge 2015), and the insulin receptor has also shown potential for enhancing brain delivery of protein drugs (Boado et al. 2014). Additionally, insulin‐like growth factor‐1 receptor (IGF1R) and low‐density lipoprotein receptor (LDLR) have been explored (Alata et al. 2022; Wang et al. 2013), along with LRP1, LRP2, and the leptin receptor (OBR) as promising mediators of CNS delivery (Liu et al. 2010; Shibata et al. 2000; Zlokovic et al. 1996).

Evaluating the brain penetration of antibody therapeutics during the preclinical stage requires a sensitive and reliable bioanalytical method to quantify antibodies in brain tissue. However, tissue analysis poses distinct challenges. Unlike plasma or serum, brain tissue contains substantial membrane components that may entrap analytes and hinder their recovery (Xue et al. 2012). For small molecules, protein precipitation with organic solvents like acetonitrile (ACN), methanol (MeOH), or isopropyl alcohol (IPA) is commonly employed (Ramalingam and Ko 2014). While protein precipitation can be effective in certain applications, it is not suitable for immunoprecipitation‐based quantification of monoclonal antibodies due to the risk of denaturation and insufficient selectivity (Magsumov et al. 2020; Zhang et al. 2018). Alternatives such as pellet formation followed by trypsin digestion may suffer from reduced sensitivity (Jiang et al. 2013; Ouyang et al. 2012) and interference from abundant endogenous peptides in LC–MS analysis (Jiang et al. 2013).

To address these limitations, several extraction strategies have been adopted in the biopharmaceutical industry to recover brain‐resident antibodies (Georgieva et al. 2020; Nakano et al. 2019; Pornnoppadol et al. 2024; Yang and Sumbria 2021; Zuchero et al. 2016). Among them, detergent‐based extraction has emerged as a preferred method due to its dual benefits: disruption of cellular and organelle membranes and protein solubilization and linearization, which enhance accessibility to proteolytic enzymes (Chen et al. 2007; Danko et al. 2022; Hjelmeland and Chrambach 1984; Moore et al. 2016; Seddon et al. 2004).

Detergents are generally classified into ionic, non‐ionic, and zwitterionic types. Ionic detergents like SDS and SDC are effective in solubilizing membrane proteins, though SDC is considered milder (Bhairi 2001; Danko et al. 2022). Zwitterionic detergents such as CHAPS efficiently disrupt protein–protein interactions with minimal denaturation (Danko et al. 2022). Non‐ionic detergents—including Tween‐20, Triton‐X, IGEPAL, and NP‐40—are widely used in immunoprecipitation assays due to their ability to preserve protein structure (Bhairi 2001; Danko et al. 2022). Additionally, chaotropic agents like urea are employed to assist solubilization but may inhibit protease activity and induce lysine carbamylation at higher concentrations (Sun et al. 2014).

While effective for extraction, detergents present obstacles in downstream analysis. During trypsin digestion, residual detergents may inhibit enzymatic activity—SDS, for instance, suppresses trypsin above 0.05%, whereas SDC is tolerable up to 10% (Lin et al. 2008; Zhou et al. 2012). Rapigest allows trypsin activity up to approximately 0.5% (Yu and Gilar 2009). In LC–MS analysis, detergents are broadly eluted during reverse‐phase (RP) chromatography, making it difficult to separate them from analytes such as peptides and thereby compromising chromatographic performance (Carr 2002). In addition, detergents interfere with electrospray ionization (ESI), leading to significant ion suppression (Annesley 2003). Therefore, detergent removal is crucial for accurate bioanalysis.

Various detergent‐removal strategies have been developed, including FASP, eFASP, S‐Trap, and SP3 (Danko et al. 2022). Although FASP is widely used and automated, it has limitations in our lab settings, including limited filter capacity, batch variability, poor filtration efficiency, and protein losses due to non‐specific binding (Wisniewski 2016). Thus, alternative methods are needed to enhance sensitivity and robustness in detergent removal (Leon et al. 2013; Wisniewski 2016).

**FIGURE 1:**
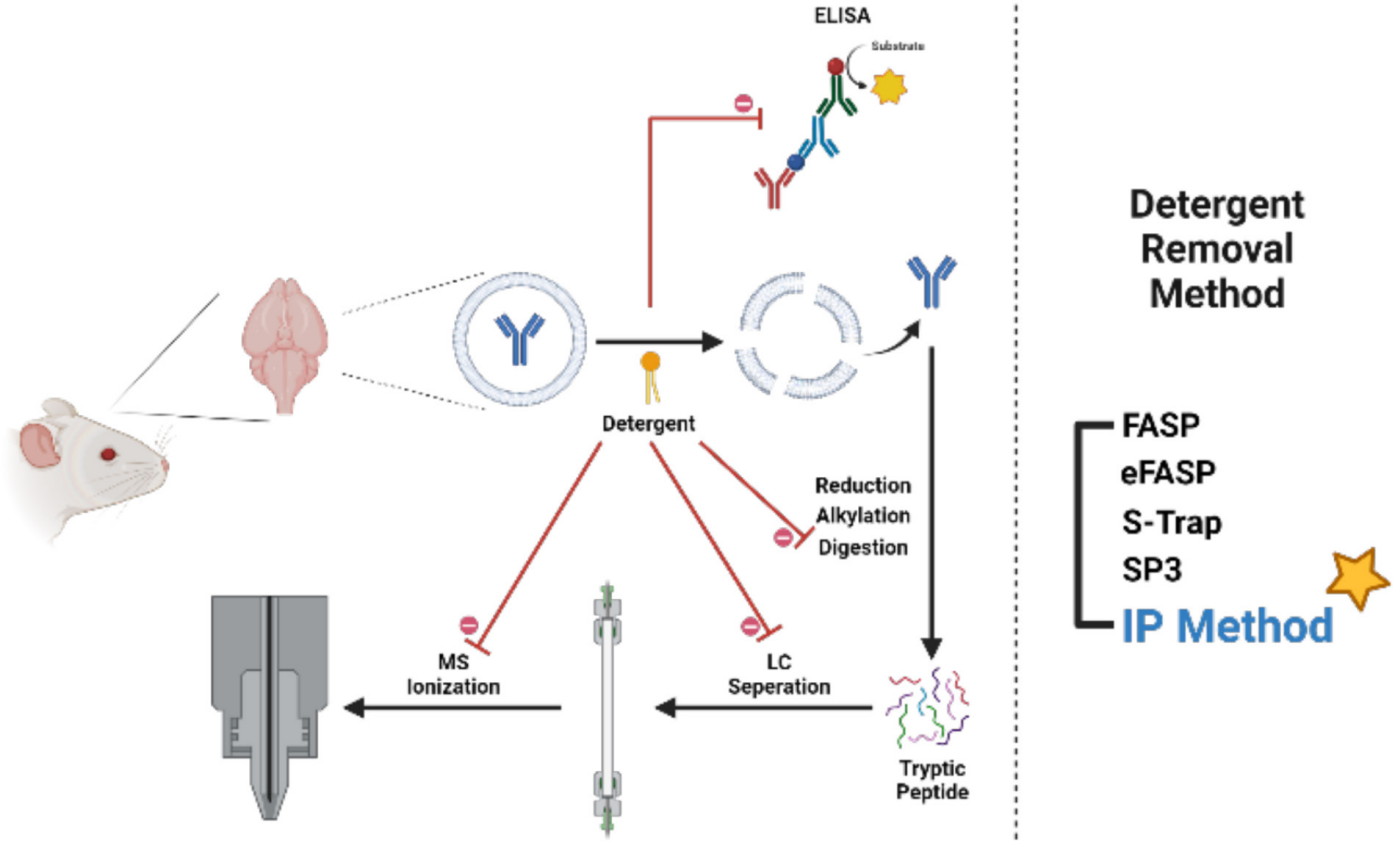
Influence of detergents on tryptic digestion and LC–MS analysis, and methods for detergent removal.

To this end, the immunoprecipitation (IP) method was selected as an enrichment approach that also removes detergents, addressing sensitivity concerns associated with low brain exposure of antibodies (Faresjo et al. 2021). Although highly sensitive assays such as ELISA could be an alternative option, these assays are less likely to function under harsh conditions suitable for sufficient cell extraction with detergents (Waritani et al. 2017). Therefore, IP was adopted as a more suitable strategy for analyte recovery and quantification under these conditions (Rosengren et al. 2003). Figure 1 illustrates the negative effects of residual detergents on proteolysis and LC–MS and compares conventional detergent removal methods with the proposed IP‐based approach.

In addition, removing blood contamination is essential in tissue analysis, as residual blood can lead to overestimation of drug concentrations (Giudicelli et al. 2004). Perfusion is widely used for this purpose (Noh et al. 2023). Although various biomarkers and tracers have been suggested to assess contamination—such as radiolabels, transferrin, and blood proteins—they may introduce bias if endogenously present in brain tissue (Grimm et al. 2023; Kang and Kho 2019; Kivlighan et al. 2004; Noh et al. 2023). As a result, hemoglobin was selected as an appropriate marker to evaluate blood contamination in brain samples (Noh et al. 2023).

In this study, we screened detergent conditions optimized for antibody extraction using the IP method and successfully developed a bioanalytical platform to quantify monoclonal antibodies in mouse brain. Using this method, we evaluated the brain penetration of antibody 8D3 in male ICR mice, a strain known for efficient BBB transcytosis via the transferrin receptor. This approach enhances our ability to assess the CNS delivery potential of therapeutic antibodies. Additionally, a hemoglobin assay was performed to determine the extent to which blood contamination influences measured antibody concentrations in brain tissue.

## 2 Materials and Methods

### 2.1 Chemical and Reagents

Trastuzumab was obtained from Samsung Medical Center (Seoul, South Korea). 8D3 was purchased from Bioxcell (NH, USA). The stable isotope‐labeled peptide, TTPP*V*LDSDGSFFLYSK (where * indicates a stable isotope‐labeled amino acid with five ^13^C and one ^15^N), used as the internal standard (ISTD) for the total antibody quantification assay, was purchased from AnyGen (Gwangju, South Korea). Protein G and protein A magnetic beads were purchased from Millipore Korea (Daejeon, South Korea). RapiGest SF was purchased from Waters Korea (Seoul, South Korea). Dithiothreitol was purchased from Carl Roth (Karlsruhe, Germany). Iodoacetic acid was purchased from Wako (Osaka, Japan). The sequencing grade modified trypsin was purchased from Promega (Wisconsin, USA). A 10× phosphate‐buffered saline (PBS) (pH 7.6) solution was purchased from LPS solution (Daedeok‐gu, Daejeon, South Korea). LC–MS‐grade ACN, distilled water (DW), and formic acid (FA) for LC‐qTOF‐MS analysis were purchased from ThermoFisher Scientific (Massachusetts, USA). HPLC‐grade DW for sample pretreatment was purchased from Samchun Chemical (Pyeongtaek, Gyeonggi, South Korea). Sodium deoxycholate (SDC), sodium dodecylsulfate (SDS), IPEGALCA‐360, cOmplete mini EDTA‐free protease inhibitor cocktail, phenylmethylsulfonyl fluoride (PMSF), and 2,2,2‐tribromoethanol (Avertin) were purchased from Sigma‐Aldrich Korea (Seoul, South Korea). Tris–HCl (1 M) (pH 8.0) was purchased from AllforLab (Seoul, South Korea). Tween‐20 and hydrochloric acid (HCl) were bought from Daejung reagents (Siheung, South Korea). Mouse hemoglobin ELISA Kit (ab254517) was purchased from Abcam (Boston, USA). All other chemicals and reagents were analytical grade and purchased from commercial sources.

### 2.2 Equipment and Liquid Chromatography–Quadrupole Time‐of‐Flight Mass Spectrometry (LC‐qTOF‐MS) Conditions

The equipment for LC‐qTOF‐MS analysis was composed of a CTC HTS PAL auto‐sampler (LEAP Technologies, Carrboro, NC, USA), two Shimadzu LC‐20ad pumps, a Shimadzu CBM‐20A HPLC pump controller (Shimadzu, Columbia, MD, USA), and a quadrupole time‐of‐flight TripleTOF 5600 mass spectrometer with a Duospray ion source (Sciex, Foster City, CA, USA). The equipment for the microplate reader was composed of a BioTek Epoch 2 microplate spectrophotometer (BioTek, Winooski, VT, USA).

The surrogate peptide derived from Trastuzumab, 8D3, and the ISTD was separated using a Phenomenex Kinetex XB‐C18 column (2.1 × 50 mm, 2.6 μm), and the temperature of the column during the separation of the two compounds was set at 55°C. The pumping mode of two Shimadzu LC‐20ad pumps was binary, and two types of mobile phases (mobile phase A; DW containing 0.1% FA, mobile phase B; ACN containing 0.1% FA) were used in the following composition (Table 1). The mobile phase B percentage of gradient elution increased from 15% mobile phase B to 95% mobile phase B during run time. The injection volume was 10 μL, and the rate of LC flow was 0.4 mL/min. After separating the surrogate peptide of Trastuzumab, 8D3, and ISTD with liquid chromatography, analytes were ionized through an electrospray ionization (ESI) source and then introduced to mass spectrometry for analysis. The temperature of the ion source was 500°C, and the voltage of the ion spray was 5500 V. The ion source gas 1 (GS1) and ion source gas 2 (GS2) were set at 50 psi, and the flow rate of curtain gas was 33 L/min.

**TABLE 1:** Mobile phase B (%) in LC gradient elution.

Time (min)	Mobile phase B (%)
0	15
0.5	15
1.4	70
1.5	95
1.7	95
1.8	15
3.0	15

The transitions of parent ion to product ion were as follows: *m/z* 937.5–836.4 for the surrogate peptide of Trastuzumab, *m/z* 649.0–359.2 for the surrogate peptide of 8D3, and *m/z* 943.5–842.4 for ISTD. The declustering potential (DP) was 162, 125, and 162 V for the surrogate peptide of Trastuzumab, 8D3, and ISTD, respectively. The collision energy (ce) was 36, 20, and 38 V for the surrogate peptide of Trastuzumab, 8D3, and ISTD, respectively.

The Analyst TF (version 1.6) and MultiQuant (version 3.0.3) software (Sciex, Foster City, CA, USA) were used for data acquisition and data processing, respectively. The GraphPad Prism (version 9.0) software (GraphPad Software, Boston, MA, USA) and BioRender (BioRender, Toronto, ON, Canada) were used for graphical data.

### 2.3 Assessment of Detergent Compatibility in Bead Binding Within the IP Method

To select an appropriate detergent and optimal dilution factors of detergents for IP methods, a detergent compatibility assessment was conducted. An appropriate detergent is one that does not interfere with antibody binding during IP and requires minimal dilution when interference is present.

A total of 4 μL of 1000‐μg/mL 8D3 in 1× PBS with 0.1% Tween‐20 was spiked to the 20 μL of mouse blank brain homogenized with basis buffer (brain weight [g]:basis buffer [mL] = 1:2). Table 2 shows the composition of the basis buffer. The detergent solution was prepared at the following concentrations: SDS (0.1%, 0.2%, 0.3%, 0.4%, and 0.5% [w/v] in 1× PBS), SDC (0.1%, 0.5%, 1%, 2%, and 5% [w/v] in 1× PBS), IGEPAL (1%, 2%, and 5% [v/v] in 1× PBS). A total of 350 μL of pre‐chilled detergent solution and 20 μL of protein G magnetic beads were added to the spiked samples. The mixture was gently shaken at room temperature on a rotary shaker for 2 h.

**TABLE 2:** Composition of the basis buffer.

Materials	Volume (μL) or amount	Final concentration (mM)	Note
DW	4150		
NaCl	300	300	
1‐M Tris–HCl	500	100	
cOmplete mini protease cocktail	One tablet		
PMSF	50	2	Add before use

Subsequently, the magnetic beads were washed once with 1× PBS containing 0.1% Tween‐20, followed by a wash with 1× PBS. The subsequent post‐IP steps were carried out following the procedure detailed in Section: Post‐IP Sample Processing and LC‐qTOF‐MS Analysis.

### 2.4 Post‐IP Sample Processing and LC‐qTOF‐MS Analysis

To prepare the captured antibody for enzymatic digestion, the washed beads were treated with 25 μL of ISTD, 75 μL of RapiGest, and 10 μL of dithiothreitol (DTT) to induce protein unfolding and reduction. After shaking for 1 min, the mixture was incubated at 60°C for 1 h. Next, 25 μL of iodoacetic acid was added to alkylate the analyte. Following another 1‐min shake, the mixture was incubated for 30 min at room temperature in a shaded environment. To digest the samples on the beads, 10 μL of sequencing‐grade modified trypsin was added. After 1 min of shaking, the mixture was incubated overnight at 37°C. To terminate the reaction, 15 μL of 2‐N HCl was introduced, and the mixture was shaken for 1 min before a 30‐min incubation at 37°C. The sample was then centrifuged at 10,000 *g* for 5 min at 4°C, and 110 μL of the supernatant was collected into an HPLC vial for LC‐qTOF‐MS analysis.

### 2.5 Evaluation of Antibody Denaturation Under Detergent Extraction Conditions

An evaluation of antibody denaturation under detergent extraction conditions was conducted to select an appropriate detergent for the extraction of antibodies from brain tissue. An appropriate detergent is a detergent that does not denature the antibody. A total of 6 μL of 100‐μg/mL 8D3 in 1× PBS with 0.1% Tween‐20 was spiked to the 30 μL of mouse blank brain homogenized with basis buffer (brain weight [g]:basis buffer [mL] = 1:2). SDS, SDC, and IGEPAL detergent solutions were prepared at concentrations of 2%, 4%, 8%, and 16% in DW. A total of 40 μL of pre‐chilled detergent solution was added to the mixture and incubated for 10, 30, and 60 min at 4°C using a rotary shaker. After incubation, the samples were centrifuged at 13,000 *g* for 5 min at 4°C, and 20 μL of supernatant was transferred to a new Eppendorf tube.

A total of 350 μL of 1× PBS with 0.1% Tween‐20 and 20 μL of protein G magnetic beads were added to the spiked samples. In the case of incubated samples under SDS conditions, to avoid potential denaturation during bead binding, 800‐μL 1× PBS with 0.1% Tween‐20 and 20 μL of protein A magnetic bead was added to the tube.

The subsequent post‐IP steps were carried out following the standard procedure detailed in Post‐IP sample processing and LC‐qTOF‐MS analysis.

### 2.6 Pharmacokinetics (PK) Study of 8D3 in Male ICR Mouse Following IV Bolus Administration at a Dose of 20 mg/kg

Male ICR mice (30 ± 3 g) were purchased from Samtako BioKorea Co. (Pocheon, Gyeonggi, South Korea) and housed in groups of 6–8 per cage and given standard rodent chow. The animals were fasted overnight with free access to water for at least 12 h before administration. Mice were distributed into five groups (*n* = 4 for each group). 8D3 was administered via a single intravenous bolus (IV) at 20‐mg/kg dose.

The blood sampling time points were 4, 8, 24, 48, and 72 h after administration. The collected blood samples were centrifuged at 13,000 rpm for 5 min, and the plasma was transferred to another tube. The samples were stored at −80°C until analysis.

Brain sampling time points were also 4, 8, 24, 48, and 72 h after administration. The brain was collected from the same individual from whom blood was drawn. The mouse was perfused transcardially with 30 mL of 1× PBS after Avertin euthanasia (Wu et al. 2021). The flow rate and perfusion time were 3 mL/min and 10 min for the mouse. The samples were snap‐freezed using liquid nitrogen and stored at −80°C until analysis.

All experiments performed on the mouse were approved, abiding by the animal care protocol (No. 202404A‐CNU‐071) from Chungnam National University. The procedures were abided by the guidelines established by the Association for Assessment and Accreditation of Laboratory Animal Care International (AAALAC International).

### 2.7 Preparation of Standard (STD), Quality Control (QC), and In Vivo Samples for the Calibration Curve of 8D3 in the Plasma Matrix

To prepare calibration STD and QC for plasma, the sub‐stock solution was serially diluted using 1× PBS with 0.1% Tween‐20. Each 4 μL of STD and QC working solution was spiked into 20 μL of mouse blank plasma. The final concentration of calibration STD concentrations was 5, 10, 20, 40, 80, 160, 320, and 400 μg/mL. The final concentrations of QC samples were 15, 60, and 240 μg/mL.

To prepare an in vivo plasma sample, 20 μL of in vivo plasma samples were mixed with 4‐μL 1× PBS with 0.1% Tween‐20. A total of 350 μL of 1× PBS with 0.1% Tween‐20 and 20 μL of protein G magnetic beads were added to the samples. The mixture was gently shaken at room temperature on a rotary shaker for 2 h. Subsequently, the magnetic beads were washed once with 1× PBS containing 0.1% Tween‐20, followed by a wash with 1× PBS. The subsequent post‐IP steps were carried out following the standard procedure detailed in Post‐IP Sample Processing and LC‐qTOF‐MS Analysis.

### 2.8 Preparation of STD, QC, and In Vivo Brain Samples in the Case of the IGEPAL Extraction Method

To prepare calibration STD and QC, the sub‐stock solution was serially diluted using 1× PBS with 0.1% Tween‐20. Each 70 μL of STD and QC working solution was spiked into 150 μL of mouse blank brain homogenized with basis buffer (brain weight [g]:basis buffer [mL] = 1:2). Pre‐chilled 4% IGEPAL solution (200 μL) was added to the mixture. The final concentrations of calibration STD were 50, 100, 200, 500, 1000, and 2000 ng/mL. The final concentrations of QC samples were 60, 300, and 1500 ng/mL.

To prepare in vivo brain samples, the samples were homogenized with basis buffer (brain weight [g]:basis buffer [mL] = 1:2). In vivo brain samples (150 μL) were mixed with 70‐μL 1× PBS with 0.1% Tween‐20. Pre‐chilled 4% IGEPAL solution (200 μL) was added to the mixture. The mixture was incubated at 4°C for 1 h using a rotary shaker; then, it was centrifuged at 13,000 *g* for 5 min at 4°C.

And 300 μL of supernatant was transferred to a new Eppendorf tube. A 20‐μL protein G magnetic bead was added to the tube. The mixture was gently shaken at room temperature on a rotary shaker for 2 h. Subsequently, the magnetic beads were washed once using 2% SDC in 1× PBS and then once using 1× PBS with 0.1% Tween‐20 and finally washed once using 1× PBS.

The subsequent post‐IP steps were carried out following the standard procedure detailed in post‐IP sample processing and LC‐qTOF‐MS analysis.

### 2.9 Preparation of STD, QC, and In Vivo Brain Samples in the Case of the SDC Extraction Method

To prepare calibration STD and QC, the sub‐stock solution was serially diluted using 1× PBS with 0.1% Tween‐20. Each 70 μL of STD and QC working solution was spiked into 150 μL of mouse blank brain homogenized with basis buffer (brain weight [g]:basis buffer [mL] = 1:2). Pre‐chilled 4% SDC solution (200 μL) was added to the mixture. The final concentrations of calibration STD were 100, 200, 1000, 2000, 5000, and 10,000 ng/mL. The final concentrations of QC samples were 300, 1500, and 7500 ng/mL.

To prepare in vivo brain samples, in vivo brain samples were homogenized with basis buffer (brain weight [g]:basis buffer [mL] = 1:2). In vivo brain homogenates (150 μL) were mixed with 70‐μL 1× PBS with 0.1% Tween‐20. Pre‐chilled 4% SDC solution (200 μL) was added to the mixture. The mixture was incubated at 4°C for 1 h using a rotary shaker; then it was centrifuged at 13,000 *g* for 5 min at 4°C.

And 300 μL of supernatant was transferred to a new Eppendorf tube. A 20‐μL protein G magnetic bead was added to the tube. The mixture was gently shaken at room temperature on a rotary shaker for 2 h. Subsequently, the magnetic beads were washed once using 2% SDC in 1× PBS and then once using 1× PBS with 0.1% Tween‐20 and finally washed once using 1× PBS.

The subsequent post‐IP steps were carried out following the standard procedure detailed in post‐IP sample processing and LC‐qTOF‐MS analysis.

### 2.10 Preparation of STD, QC, and In Vivo Brain Samples in the Case of Control Extraction Method

To prepare calibration STD and QC, the sub‐stock solution was serially diluted using 1× PBS with 0.1% Tween‐20. Each 70 μL of STD and QC working solution was spiked into 150 μL of mouse blank brain homogenized with basis buffer (brain weight [g]:basis buffer [mL] = 1:2). Pre‐chilled protease inhibitor cocktail in DW (200 μL) was added to the mixture. The final concentrations of calibration STD were 100, 500, 1000, 2000, 5000, and 10,000 ng/mL. The final concentrations of QC samples were 300, 1500, and 7500 ng/mL.

To prepare in vivo brain samples, in vivo brain samples were homogenized with basis buffer (brain weight [g]:basis buffer [mL] = 1:2). In vivo brain samples (150 μL) were mixed with 70‐μL 1× PBS with 0.1% Tween‐20. Pre‐chilled protease inhibitor cocktail solution (200 μL) was added to the mixture. The mixture was incubated at 4°C for 1 h using a rotary shaker; then, it was centrifuged at 13,000 *g* for 5 min at 4°C.

And 300 μL of supernatant was transferred to a new Eppendorf tube. Protein G magnetic bead (20 μL) was added to the tube. The mixture was gently shaken at room temperature on a rotary shaker for 2 h. Subsequently, the magnetic beads were washed once using 2% SDC in 1× PBS and then once using 1× PBS with 0.1% Tween‐20 and finally washed once using 1× PBS.

The subsequent post‐IP steps were carried out following the standard procedure detailed in post‐IP sample processing and LC‐qTOF‐MS analysis.

### 2.11 Analysis of PK Study Results

PK parameters were calculated through non‐compartmental analysis (NCA) using Phoenix WinNonlin software (version 8.1; Pharsight Corporation, Mountain View, CA, USA), and the PK parameters of the individual mice were determined. Also, the mean values of PK parameters for each dosing group were determined. The analysis of the PK study included extrapolated concentrations higher than 50% of the lower limit of quantification (LLOQ).

Among the various PK parameters, representative PK parameters were reported in this paper; the observed maximum plasma concentration (C_max_) and total drug exposure were defined as the area under the concentration–time curve to the last time point (AUC_last_).

### 2.12 Hemoglobin Quantification in the Perfused and Non‐Perfused Brain

The perfused brain samples were prepared in the following steps. The mouse was perfused transcardially with 1× PBS after Avertin euthanasia (Wu et al. 2021). The flow rate and perfusion time were 3 mL/min and 10 min for the mouse. The non‐perfused brain samples were prepared in the following steps. The mouse was cardiac punctured, and then the brain was collected carefully using forceps. Brain samples were homogenized with 1× cell extraction buffer PTR (brain weight [g]:cell extraction buffer PTR [mL] = 1:10). Perfused and non‐perfused brain homogenates were diluted 810 and 65,610 times, respectively, using 1× cell extraction buffer PTR. ELISA was conducted by user instruction (Cat No. ab254517).

## 3 Results

### 3.1 Assessment of Detergent Compatibility in Bead Binding Within the IP Method

**FIGURE 2:**
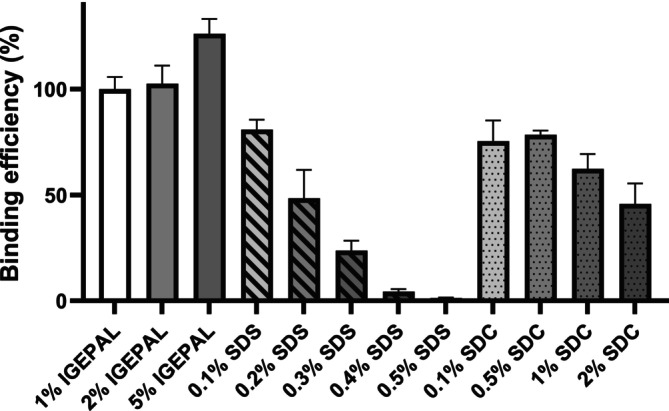
Binding efficiency (%) of the antibody under the various detergent conditions after bead binding for 2 h at RT (the binding efficiency [%] was calculated by assuming the area ratio value of the 1% IGEPAL sample as 100%, *N* = 3).

A key factor in the IP technique for quantifying antibodies is ensuring effective antibody binding by protein A or G beads. Detergents play a significant role in the IP process through two main mechanisms, one of which includes possible interference with bead binding, potentially disrupting the interaction between antibodies and protein A or G beads (DeCaprio and Kohl 2020; Nøkleby et al. 2008). To identify a detergent compatible with the IP technique, this study examined how detergent type and concentration affected bead binding efficiency. A control sample under mild conditions—1% IGEPAL solution served—as our baseline for calculating the percentage of antibodies remaining intact. The primary consideration in selecting a suitable detergent was its ability to maintain consistent binding efficiency (%) even at increasing concentrations. Figure 2 presents the bead binding efficiency (%) across various detergent conditions following bead binding. Typical concentration ranges for IP‐compatible detergents are up to 0.1% for ionic detergents and up to 1% for non‐ionic detergents. This investigation, however, extended beyond these conventional thresholds to evaluate binding efficiency at higher concentrations, prompted by concerns that typical IP buffer conditions might inadequately extract target proteins.

SDS, an ionic detergent, showed a significant decline in binding efficiency as concentrations increased. Binding efficiency (%) at SDS concentrations of 0.1%, 0.2%, 0.3%, 0.4%, and 0.5% were 80.9% ± 4.6%, 48.4% ± 13.5%, 23.7% ± 4.6%, 4.4% ± 1.2%, and 1.3% ± 0.3%, respectively. These findings indicate that SDS is unsuitable for IP applications above typical concentrations.

On the other hand, IGEPAL, a non‐ionic detergent, demonstrated exceptional compatibility even at concentrations surpassing the conventional 1% limit. Binding efficiency (%) at 1%, 2%, and 5% IGEPAL was 100.0% ± 5.8%, 102.6% ± 8.6%, and 126.2% ± 7.0%, respectively, with no significant impact on binding efficiency across increasing concentrations. Similarly, SDC, another ionic detergent, showed significantly better binding efficiency compared to SDS. Additionally, there were no major changes observed when the concentration was increased by 5 to 10 times, within the range of 0.5% to 1%. Binding efficiency (%) values at 0.1%, 0.5%, 1%, and 2% SDC were 75.5% ± 9.7%, 78.5% ± 1.9%, 62.3% ± 7.0%, and 45.8% ± 9.8%, respectively. However, at 5% SDC, the magnetic beads lost their magnetism, preventing reliable data collection. Despite this limitation, SDC exhibited consistent performance at higher concentrations than typical concentrations, supporting its potential use in IP methods involving higher detergent exposure.

To validate these observations, further experiments were conducted with Trastuzumab (Data S1). The findings aligned with the primary results. IGEPAL consistently showed minimal impact on binding efficiency across increasing concentrations, while SDC maintained stable patterns. In contrast, SDS experienced a pronounced decrease in binding efficiency above 0.3%, reaffirming its unsuitability for higher detergent levels.

In summary, IGEPAL and SDC emerged as effective detergents for IP applications involving elevated concentrations, with IGEPAL showing the highest level of compatibility. Conversely, SDS is unsuitable due to its marked decline in binding efficiency beyond conventional limits. These results emphasize the need to optimize detergent selection in IP workflows.

It is worth noting that detergents may not only affect the structure of the analyte antibody but also potentially disrupt the Fc‐binding functionality of Protein A or G immobilized on magnetic beads. The current assay design enables us to distinguish these possibilities, as the IP binding efficiency results reflect the bead‐binding compatibility. These findings thus support the interpretation that there is detergent‐induced interference.

### 3.2 Evaluation of Antibody Denaturation Under Detergent Extraction Conditions

**FIGURE 3:**
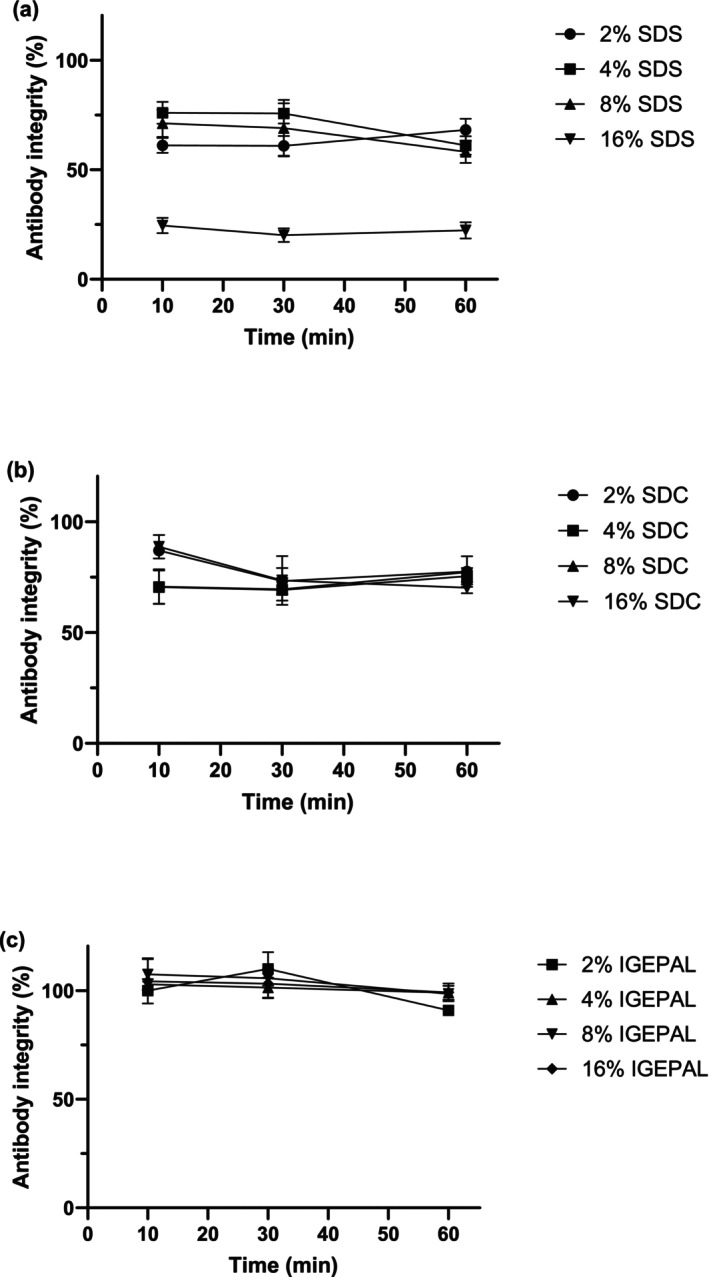
Antibody integrity (%) under (a) SDS conditions, (b) SDC conditions, and (c) IGEPAL extraction conditions at 4°C (the antibody integrity [%] was calculated by assuming the area ratio value of the 2% IGEPAL, 10‐min incubation sample as 100%, *N* = 3).

Another factor to consider is the detergent‐induced denaturation of antibodies, which can affect their three‐dimensional structure during tissue extraction, thereby preventing interaction with protein A or G beads (Desfougeres et al. 2016; Nøkleby et al. 2008). To assess the extent of antibody denaturation with detergent use, we performed denaturation tests. A control sample under mild conditions—2% IGEPAL solution with a 10‐min incubation—served as our baseline for calculating the percentage of antibodies remaining intact. After a 60‐min incubation, we compared the area ratio at SDS, SDC, and IGEPAL at 2%, 4%, 8%, and 16% incubation conditions to the baseline and area ratio value of 2% IGEPAL. Figure 3 illustrates the variation in antibody integrity (%) over time under different conditions for each detergent. Our findings showed that at concentrations of 2%, 4%, 8%, and 16% after 60 min of incubation, SDS resulted in antibody integrity (%) of 68.3% ± 5.1%, 61.1% ± 4.3%, 58.2% ± 5.2%, and 22.4% ± 3.7%, respectively, indicating that SDS can denature antibodies. Conversely, SDC showed antibody integrity (%) of 77.5% ± 7.0%, 75.4% ± 3.7%, 77.3% ± 1.9%, and 70.2% ± 2.5% at concentrations of 2%, 4%, 8%, and 16%, respectively. Similarly, IGEPAL yielded antibody integrity (%) of 90.9% ± 2.3%, 99.1% ± 3.3%, 98.6% ± 2.3%, and 99.2% ± 4.2% at 2%, 4%, 8%, and 16% concentrations. Both SDS and SDC did not exhibit antibody denaturation within 10–60 min of the experiment. Despite low detergent concentrations due to extensive dilution, residual SDS and SDC affected bead binding, as depicted in Figure 2, leading to a reduction in the measured antibody integrity (%). Specifically, SDS showed significant reductions in antibody integrity (%) only at concentrations exceeding 16%. In contrast, IGEPAL demonstrated no evidence of time‐dependent denaturation. When the experiment was replicated using a Trastuzumab, no time‐dependent denaturation was observed with SDC after 10 min, aligning with the results for IGEPAL (Data S2). However, SDS displayed a slight variation, showing time‐dependent denaturation at a concentration of 4% in this antibody. These findings indicate that IGEPAL and SDC are broadly suitable for use in the IP method.

### 3.3 PK Study of 8D3 in Male ICR Mouse

**FIGURE 4:**
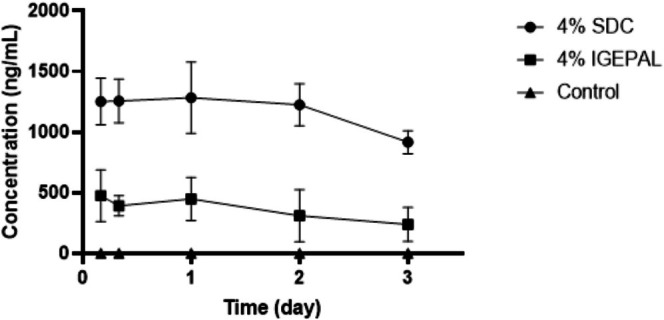
Mean time–concentration profile of 8D3 in mouse brain following IV bolus administration at a dose of 20 mg/kg using 4% IGEPAL, 4% SDC, control extraction method (*N* = 4).

**FIGURE 5:**
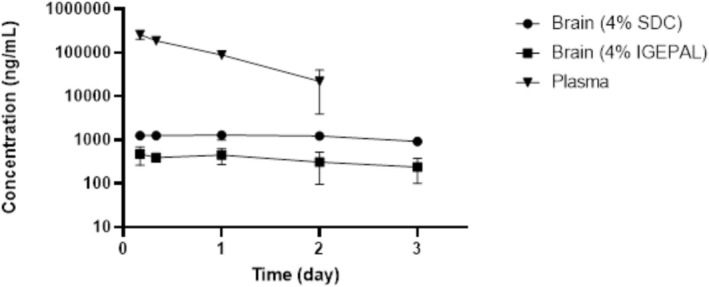
Mean time–concentration profiles of 8D3 in mouse plasma and brain following IV bolus administration at a dose of 20 mg/kg (*N* = 4).

Plasma samples from male ICR mice in the 8D3 pharmacokinetic (PK) study were analyzed after a single intravenous (IV) bolus administration at a dose of 20 mg/kg. Brain samples from the same study were extracted using 4% SDC and 4% IGEPAL methods to evaluate the impact of detergents on extraction efficiency; additional extractions were performed using a detergent‐free buffer as a control, followed by analysis. For the bioanalysis of PK samples in this exploratory study, fit‐for‐purpose bioanalytical methods were carried out with various concentrations of STD and QC samples at three different levels of concentrations using LC‐qTOF‐MS. The detailed information on LLOQ, upper limit of quantitation (ULOQ), and QC concentrations is described in the experimental method section. The in‐house bioanalytical acceptance criteria for STD and QC samples in the bioanalytical runs were within ±25% of the nominal values for the bioanalytical accuracy, which is also typical acceptance criteria for discovery stage studies in the biopharmaceutical industry (Bateman et al. 2013). All bioanalytical runs passed the in‐house acceptance criteria with the correlation of coefficient for all calibration curves greater than 0.99 (data not shown). Figure 4 illustrates the mean brain time‐concentration profiles of 8D3 obtained with both extraction methods, as well as the control method. Figure 5 compares the mean time‐concentration profiles of 8D3 in the mouse brain and plasma using both extraction methods. Pharmacokinetic (PK) parameters were obtained using Phoenix WinNonlin software, and individual mouse PK parameters were determined. Additionally, the mean PK parameters for both plasma and brain were calculated and are presented in Table 3. For 8D3 administered at 20 mg/kg, the observed maximum concentration (C_max_) in plasma was 258.53 μg/mL, with an area under the curve (AUC_last_) of 235.70 day*μg/mL. In the brain, using 4% IGEPAL extraction, the C_max_ was 0.48 μg/mL, and the AUC_last_ was 1.01 day*μg/mL. With 4% SDC extraction, the C_max_ was 1.28 μg/mL, and the AUC_last_ was 3.49 day*μg/mL. The brain‐to‐plasma ratio was calculated as follows.

$$\text{Brain}-\text{to}-\text{plasma ratio}\;\left(\%\right)=\left(\frac{{\mathrm{AUC}}_{\text{last}\;\left(\text{Brain}\right)}}{{\mathrm{AUC}}_{\text{last}\;\left(\text{Plasma}\right)}}\right)\times 100\;\left(\%\right)$$

**TABLE 3:** PK parameters in plasma and brain after the administration of 8D3 in ICR mouse following IV bolus administration at a dose of 20 mg/kg.

Matrix	Extraction method	C_max_ (μg/mL)	AUC_last_ (day*μg/mL)	Brain‐to‐plasma ratio (%)
Plasma		258.53	235.70	
Brain	4% IGEPAL	0.48	1.01	0.43
	4% SDC	1.28	3.49	1.48

Using 4% IGEPAL and 4% SDC extractions, the brain‐to‐plasma ratios were 0.43% and 1.48%, respectively. These findings highlight the importance of detergent selection for accurate protein extraction and analyte distribution in brain tissue. The 4% SDC method, in particular, appears to enhance the brain‐to‐plasma ratio effectively.

### 3.4 Hemoglobin Quantification in the Perfused and Non‐Perfused Brain

**FIGURE 6:**
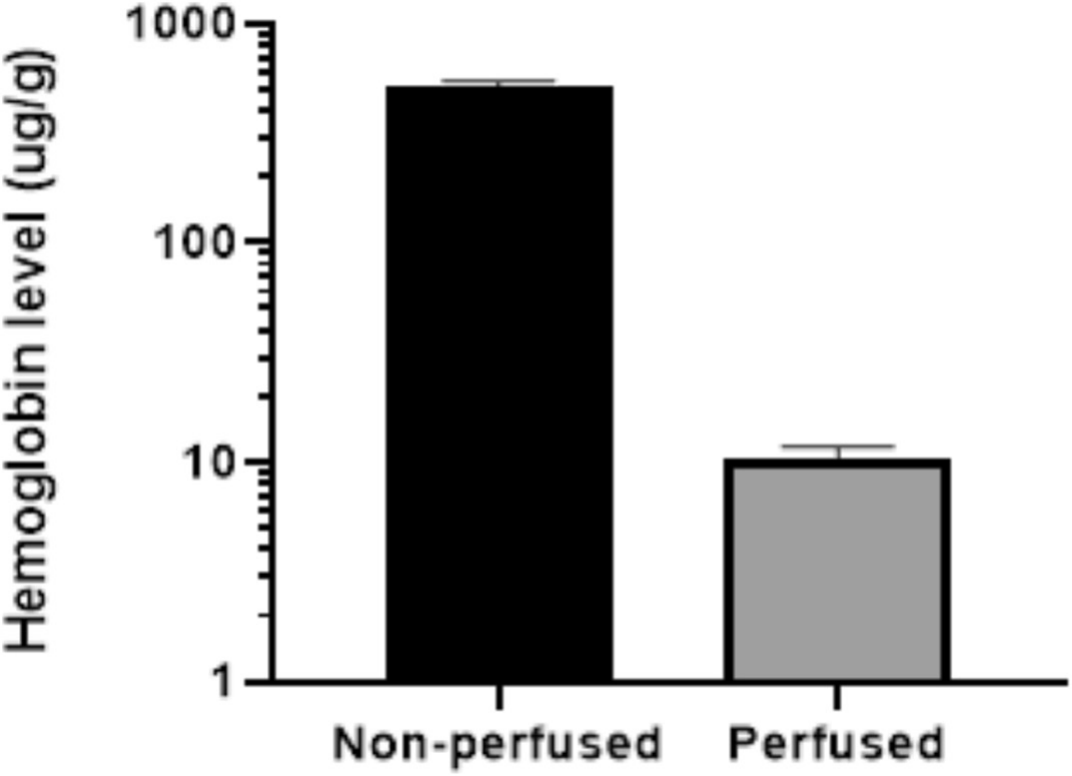
Hemoglobin level in the non‐perfused and perfused brain (*N* = 4).

An ELISA for mouse hemoglobin was conducted to ascertain the level of blood contamination in brain samples. This assay aimed to determine if the presence of blood influenced the 4% SDC extraction method. According to Figure 6, there was a marked difference in hemoglobin content between non‐perfused and perfused brain tissues. The hemoglobin concentration in non‐perfused brains was 509.40 ± 35.16 μg/g, while in perfused brains, it significantly dropped to 10.26 ± 1.48 μg/g. This denotes a residual hemoglobin content of just 2.01% relative to the non‐perfused tissue.

Given the estimated blood volume of 20 μL in the mouse brain, it was possible to estimate the total antibody content in the blood of the non‐perfused brains by using the antibody concentration found in plasma (Chugh et al. 2009). The residual antibody concentration in the perfused brain tissue, attributable to blood contamination, was determined by applying a factor of 2.01% to the antibody concentration measured in plasma. By comparing this with the actual concentration of antibodies within the brain tissue, we established a ratio.

For samples processed with the 4% SDC method, this ratio fell within the accepted range of experimental accuracy, which is 25%, in‐house criteria. These findings suggest that the 4% SDC extraction method is sufficiently robust to significantly reduce blood contamination, particularly when employing a perfusion protocol of 3 mL/min for 10 min.

## 4 Conclusions

In conclusion, our thorough investigation has led to significant advancements in antibody extraction methods in IP using optimized detergent conditions. We have identified detergents compatible with IP—IGEPAL and SDC—that ensure minimal antibody denaturation during brain tissue processing.

Pharmacokinetic analysis of the 8D3 monoclonal antibody, administered at 20 mg/kg, revealed that the brain exposure achieved with the 4% IGEPAL and SDC extraction methods was significantly higher compared to detergent‐free methods. Among the extraction methods, the extraction method using 4% SDC was superior.

This finding also highlights the critical role of proper detergent selection in analyzing antibody distribution in the brain. Finally, our study verified that the 4% SDC extraction method did not overestimate brain exposure due to blood contamination, with a residual hemoglobin level of just 2.01% in perfused brain tissue. These insights pave the way for the refined analysis of brain‐targeted antibodies in preclinical research, emphasizing the need for methodological precision in the study of their pharmacokinetics and distribution.

## Conflicts of Interest

The authors declare no conflicts of interest.

## Supporting information


**Data S1:** Binding efficiency (%) of the under the various detergent conditions after bead binding for 2 h at RT (the binding efficiency [%] was calculated by assuming the area ratio value of the 1% IGEPAL sample as 100%, *N* = 3).
**Data S2:** Antibody integrity (%) under (a) SDS conditions, (b) SDC conditions, and (c) IGEPAL extraction conditions at 4°C (the antibody integrity [%] was calculated by assuming the area ratio value of the 2% IGEPAL, 10 min incubation sample as 100%, *N* = 3).
**Data S3‐1:** Mass‐to‐charge ratio (*m*/*z*) of the 8D3 surrogate peptide (SPQLLIYGATSLADGVPSR) obtained after immunoprecipitation and enzymatic digestion with trypsin.
**Data S3‐2:** Mass‐to‐charge ratio (*m*/*z*) of the 8D3 surrogate peptide (SPQLLIYGATSLADGVPSR) obtained after immunoprecipitation and enzymatic digestion with trypsin.
**Data S3‐3:** MS/MS spectrum of the 8D3 surrogate peptide acquired under optimized declustering potential (DP) and collision energy (ce) conditions; precursor *m*/*z* is not shown due to complete fragmentation.

## Data Availability

The datasets used and/or analyzed during the current study are available from the corresponding author on reasonable request.
