# Supplementary material for: Development of a Novel Immunoprecipitation Method for Extracting Monoclonal Antibodies From Brain Tissue and Its Application to Assessing In Vivo Brain Penetration in Mouse via Liquid Chromatography–Mass Spectrometry
Source: Biomed Chromatogr. 2025 Aug 14;39(9):e70196. doi: 10.1002/bmc.70196 (PMC12351365; doi:10.1002/bmc.70196)
Supplement: Supplementary file 1 — Data S1: Binding efficiency (%) of the under the various detergent conditions after bead binding for 2 h at RT (the binding efficiency [%] was calculated by assuming the area ratio value of the 1% IGEPAL sample as 100%, N = 3). Data S2: Antibody integrity (%) under (a) SDS conditions, (b) SDC conditions, and (c) IGEPAL extraction conditions at 4°C (the antibody integrity [%] was calculated by assuming the area ratio value of the 2% IGEPAL, 10 min incubation sample as 100%, N = 3). Data S3‐1: Mass‐to‐charge ratio (m/z) of the 8D3 surrogate peptide (SPQLLIYGATSLADGVPSR) obtained after immunoprecipitation and enzymatic digestion with trypsin. Data S3‐2: Mass‐to‐charge ratio (m/z) of the 8D3 surrogate peptide (SPQLLIYGATSLADGVPSR) obtained after immunoprecipitation and enzymatic digestion with trypsin. Data S3‐3: MS/MS spectrum of the 8D3 surrogate peptide acquired under optimized declustering potential (DP) and collision energy (ce) conditions; precursor m/z is not shown due to complete fragmentation. [file BMC-39-e70196-s001.docx]

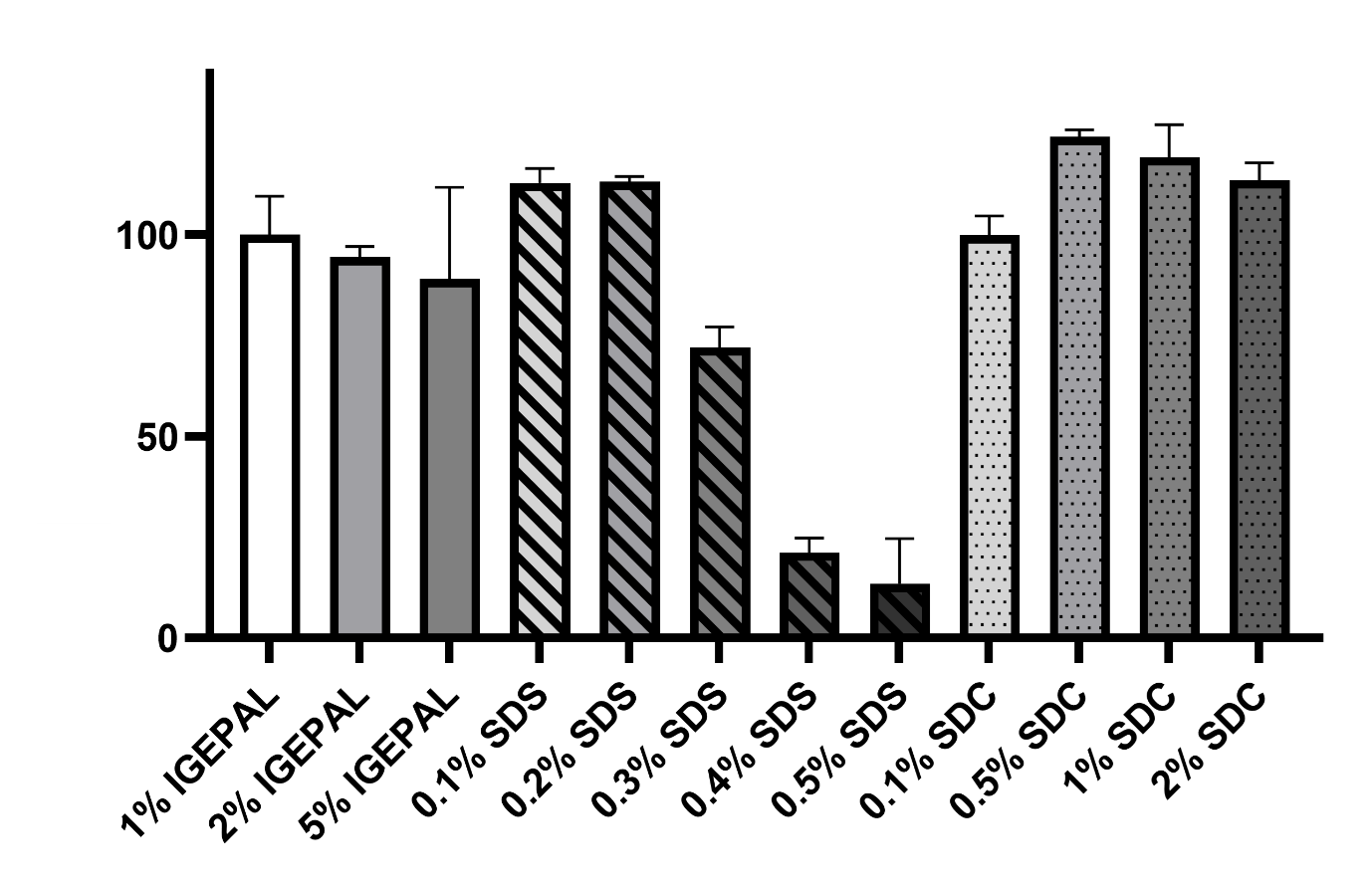


**Supplementary data S1.** Binding efficiency (%) of the under the various detergent conditions after bead binding for 2 hours at RT. (The binding efficiency [%] was calculated by assuming the area ratio value of the 1% IGEPAL^®^ sample as 100%, N=3)


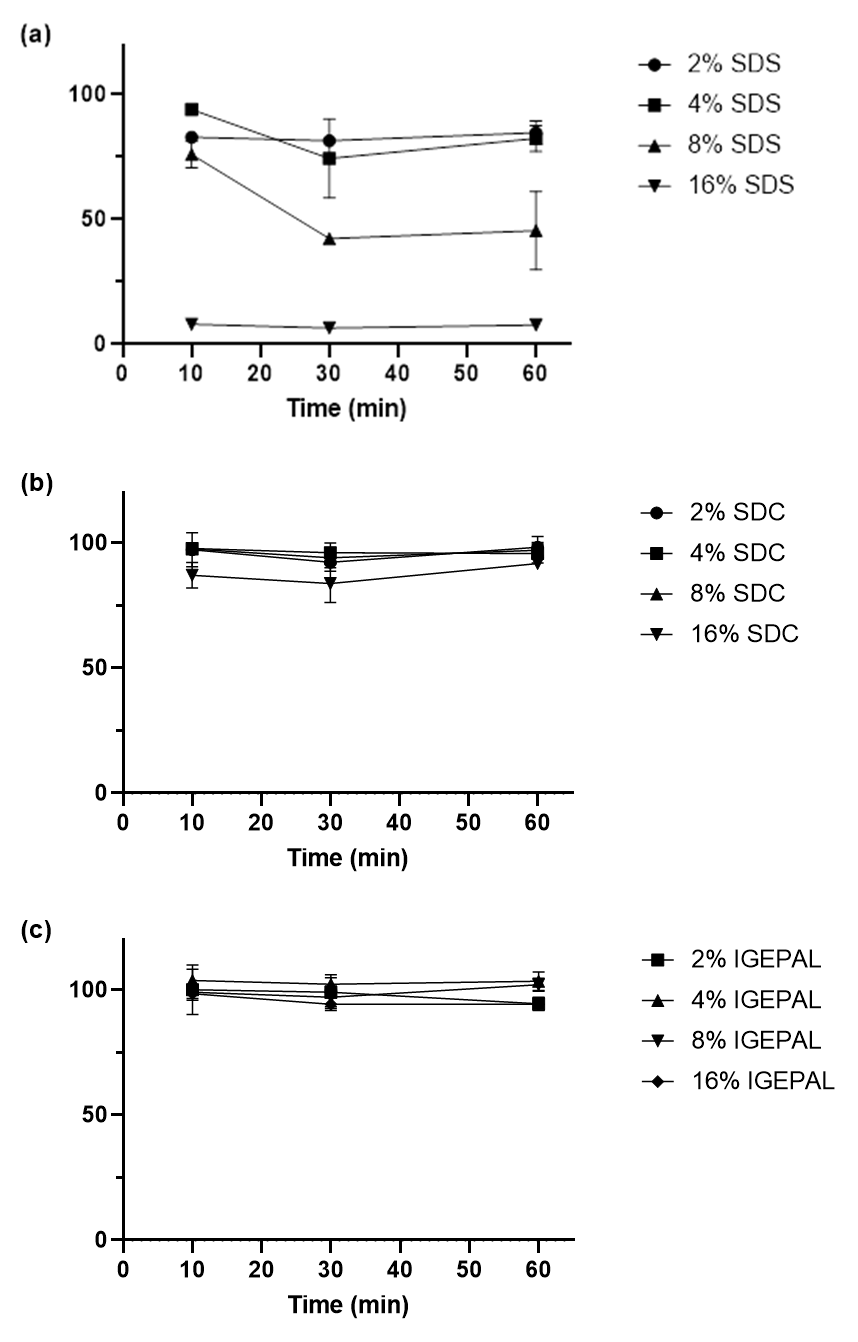


**Supplementary data S2.** Antibody integrity (%) under (a) SDS conditions, (b) SDC conditions, and (c) IGEPAL^®^ extraction conditions at 4°C. (The antibody integrity [%] was calculated by assuming the area ratio value of the 2% IGEPAL^®^, 10 minutes incubation sample as 100%, N=3)


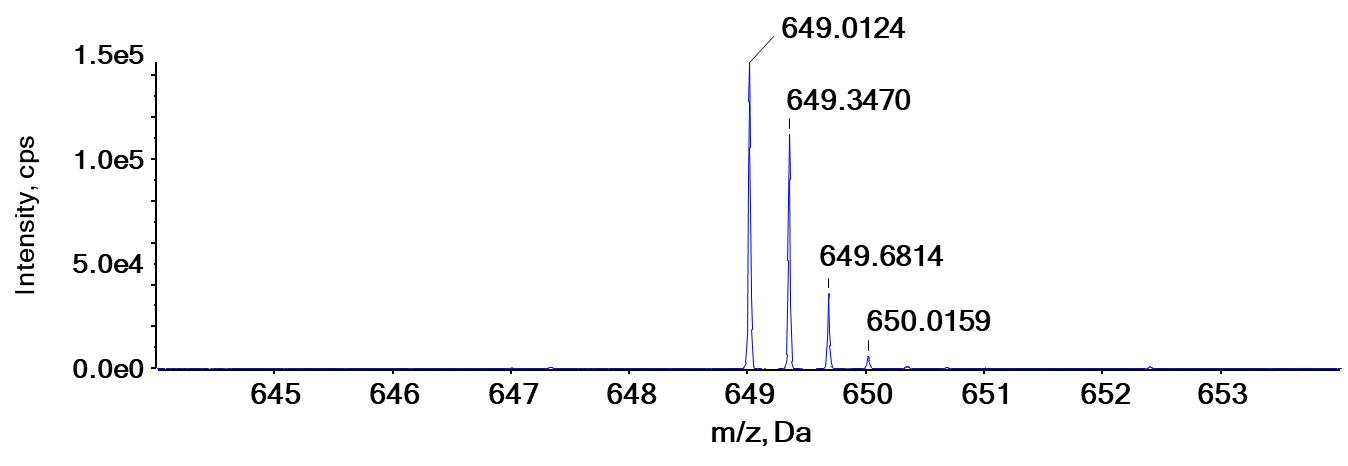


**Supplementary data S3-1.** Mass-to-charge ratio (m/z) of the 8D3 surrogate peptide (SPQLLIYGATSLADGVPSR) obtained after immunoprecipitation and enzymatic digestion with trypsin


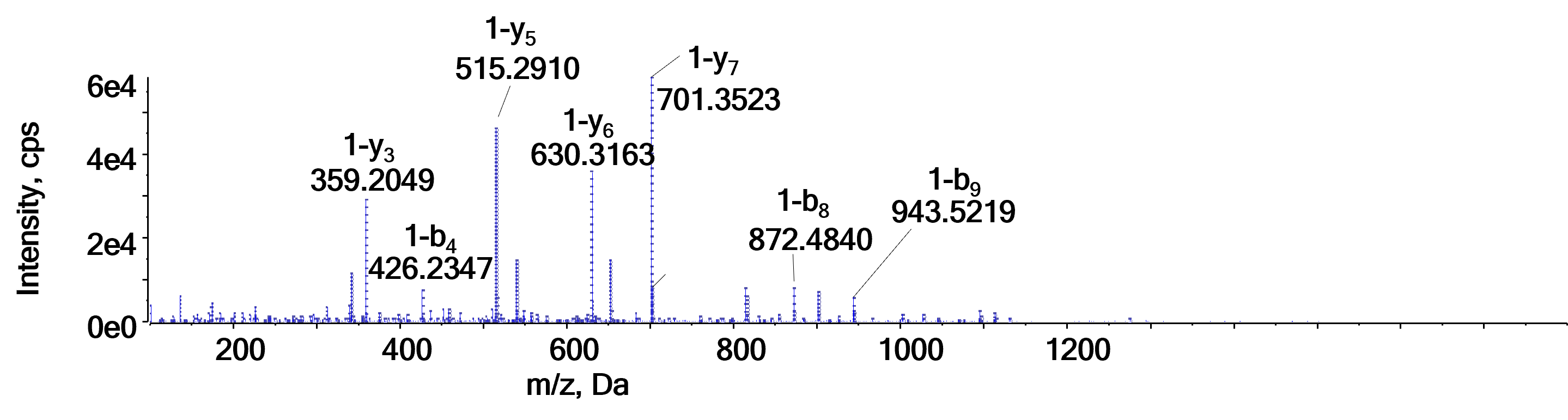


**Supplementary data S3-2.** MS/MS spectrum of the 8D3 surrogate peptide (m/z 649.0124) acquired using the information-dependent acquisition (IDA) method


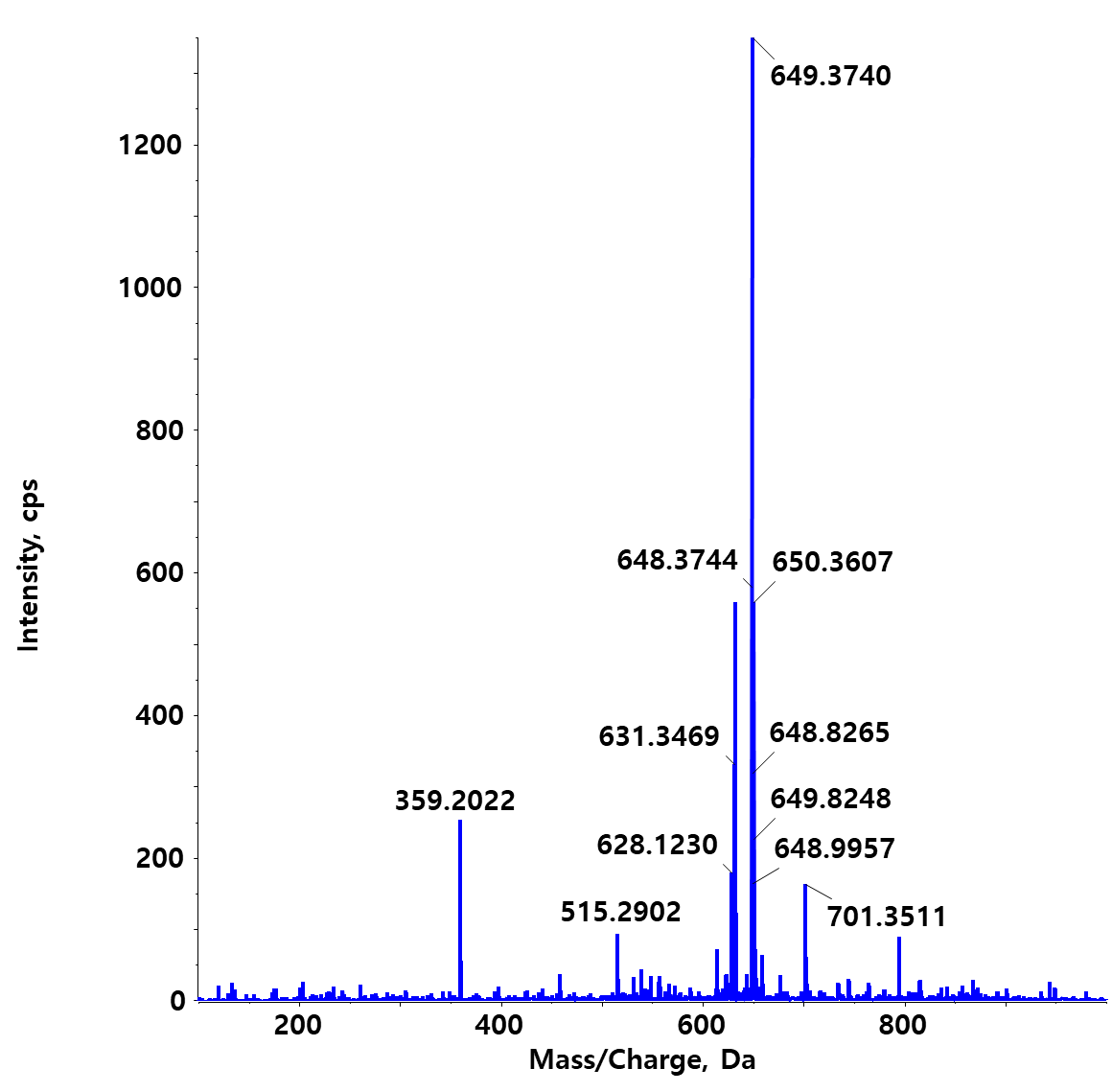


**Supplementary data S3-3.** MS/MS spectrum of the 8D3 surrogate peptide acquired under optimized declustering potential (DP) and collision energy (CE) conditions; precursor m/z is not shown due to complete fragmentation
